# Supplementary material for: ImmunoNX: a robust bioinformatics workflow to support personalized neoantigen vaccine trials
Source: ArXiv. 2025 Dec 9:arXiv:2512.08226v1. Preprint. [Version 1] (PMC12709488)
Supplement: Supplement 1 [file NIHPP2512.08226v1-supplement-1.pdf]

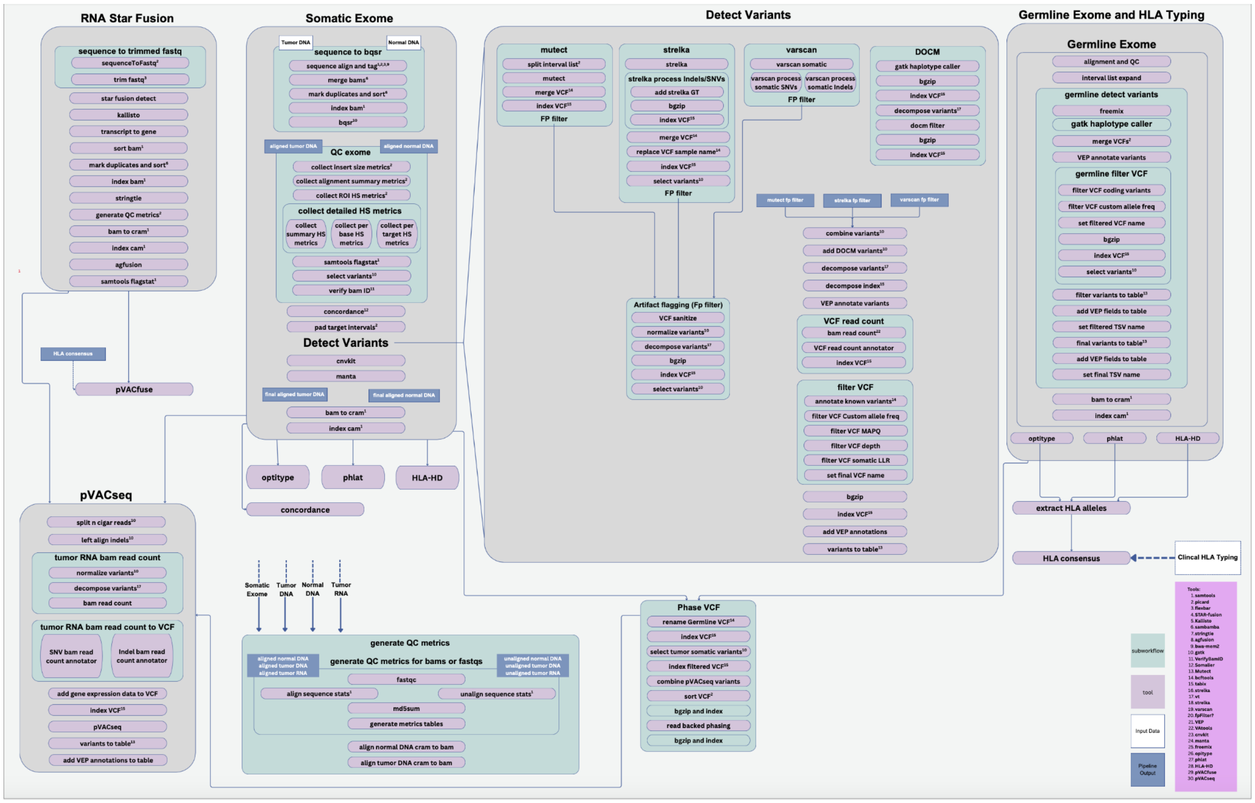

Supplementary Figure 1: Tools used in the ImmunoNX pipeline. The primary WDL that runs the ImmunoNX pipeline comprises multiple workflows such as ‘Germline Exome and HLA Typing’, ‘Somatic Exome’, etc. depicted in the large rectangles. Each workflow includes subworkflows depicted in green, that in turn run tasks or tools depicted in purple.

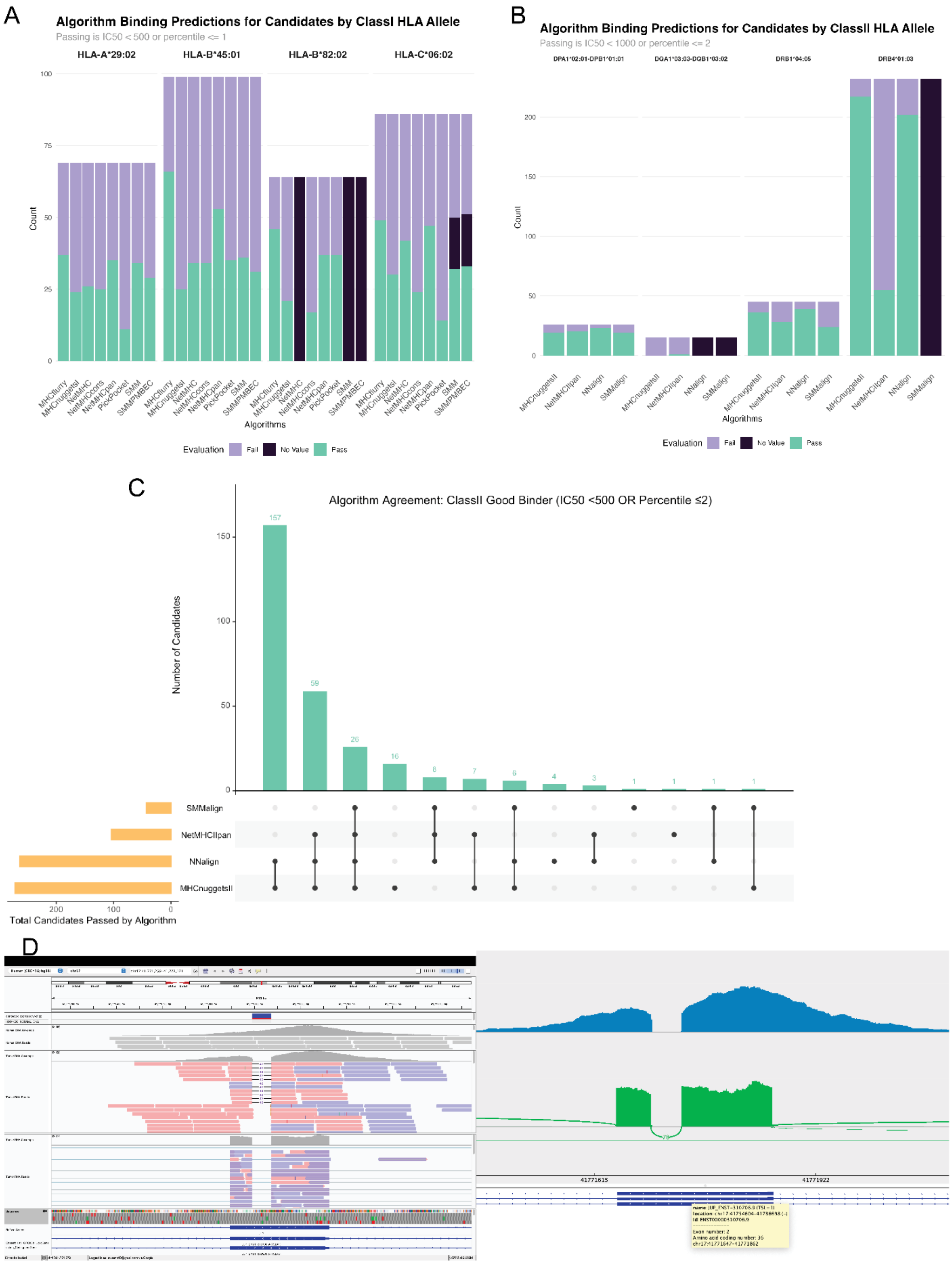

Supplementary Figure 2: Neoantigen prediction workflow results for HCC1395. **(A)** Class I and **(B)** Class II Bar charts grouped by HLA alleles showing each algorithm's binding predictions. Stacks within each bar show the number of candidates that were below the binding thresholds ('Pass' in green), above binding thresholds ('Fail' in lavender),
